# Working Women in Large Towns

**Published:** 1890-09-20

**Authors:** 


### Working Women in Large Towns.

X.—DOMESTIC SERVANTS.—(*Continued.*)

In our last article, we discussed the indoor life of the "slavey." As for her much-coveted holidays and "afternoons out," it is by means of these that many a little child-servant is inducted into the downward path. She has no friends to go to; she is young, ignorant, and longing for a little amusement; and her mistress does not trouble herself as to how she spends her holiday, only caring—if she is strict in her ways—that the girl shall be in at the time named. So the little slavey amuses herself in any way that turns up, often going with bad companions, and perhaps coming back an hour or two late to find the doors shut against her, and herself left to spend the night where she can. Or again, she may be dismissed from her place at an hour's notice for some fault or misdemeanour, and find herself suddenly homeless, placeless, friendless. What must be the risks of such a position? True, it can only be bad mistresses who would treat a girl in this way, but there are, alas! many such in our land; and no one, we think, will maintain that it is natural depravity alone which makes so many of these girls go wrong. Some years ago, when Mrs. Nassau Senior took up the cause of the little workhouse-school girls, and tried to trace out their after career, she found that the average of those who had come to utter disaster was something cruel. And, although much has been done for servant girls since then, we still hear from those who have inquired into the matter that prostitution mostly takes its recruits from the class of young servants. America, too, tells a sad tale. Out of nearly four thousand prostitutes who were questioned by the agents of the Commissioner of Labour, no less than eleven hundred proved to have been engaged in "house-work, hotel-work, table-work, and cooking." When we consider that about the same number had had "no previous occupation," the number of servants, as compared with that of women engaged in other industries, is at once seen to be painfully large.

It cannot be domestic service *in se* which is injurious. In fact, we are of opinion that it is, or ought to be, the safest and best employment for young women that can be found. To come under the civilising influences of a family above her in social rank should surely be a most valuable thing for a "daughter of the people." If there is anything in education, it should make women better housekeepers, better wives and mothers, better members of the community at large, than the mother of the slavey is likely to be. Lessons should be learnt and habits formed in such a "place," which may serve the girl in good stead when she in her turn becomes a housekeeper, a wife, and a mother. If this is not the result of her "service," it must be the fault of the mistress as well as of herself.

But a word or two must be given to a class of young servants which has as yet been barely mentioned—those girls who go straight from their London homes into service. Some of them only become "day-maids," sleeping at home, and much valuing the liberty which they can in this way secure when the daily work is over. But many others go into "places"; and a good handful will one of these servants often prove to her mistress. She is the very opposite of the little district-school girl—bright, sharp, conversant with all the ways of the world (as she knows it), fond of fun and finery, up to all sorts of tricks, and, if sharp at her work, equally sharp in devices for shirking it. It is hardly to be wondered at that mistresses regard such servants as heavy crosses in their lot. Yet even these girls are susceptible to kindness; and the treatment which they receive at the hands of their mistresses will do much to make or mar them. We dwell at some length on this aspect of the subject, because we believe that domestic service, being the only employment which really prepares a girl for the fulfilment of the duties that come later on in life, should be rendered more attractive, more useful, and more safe, by all means in our power. That girls should look upon domestic service as dull and monotonous drudgery is hardly wonderful; but the bright, happy faces of the servants in houses where the mistress rules with kindness, sympathy, and wisdom are standing evidence that service need not be dull and uninviting. These girls do, in fact, but exchange a home that perhaps was dull, squalid, and bare for another which is full of comfort, life, and varied interests. Even arduous work will be cheerfully undertaken by a servant who has learnt to consider herself a part of the family. "We've got company to dinner," she will say; or, "We can't let that dear boy go off to school without his cake and tarts—not if I sit up all night for it"; and that "we" will help her through many a toil, many a trouble. "Put it down a We, my lord, put it down a We," has more applications than Mr. Tony Weller, with all his sagacity, was aware of at the time.

*(To be continued.)*

